# Utilizing BD MAX™ Enteric Bacterial Panel to Detect Stool Pathogens from Rectal Swabs

**DOI:** 10.1186/s12907-017-0043-2

**Published:** 2017-04-08

**Authors:** Barbara DeBurger, Sarah Hanna, Eleanor A. Powell, Cindi Ventrola, Joel E. Mortensen

**Affiliations:** Cincinnati Children’s Hospital Medical Center, 3333 Burnet Avenue, Cincinnati, OH 45229 USA

**Keywords:** BDM-, Diarrhea, Bacterial stool pathogens, Rectal swabs

## Abstract

**Background:**

The BD MAX™ Enteric Bacterial Panel (BDM-EBP) is designed and FDA-cleared to detect *Salmonella, Shigella, Campylobacter,* and Shiga toxin genes *stx1/2* from stool samples. However, rectal swabs, which are not FDA-cleared for clinical testing with the BDM-EBP, are common specimens received from pediatric patients for enteric pathogen testing. The purpose of this study was to evaluate the ability of the BDM-EBP to detect stool pathogens from rectal swabs.

**Methods:**

Routine cultures, Shiga toxin testing, and molecular testing with BDM-EBP were performed on 272 sequential rectal swabs collected from August 2015 to December 2015. Discrepant test results were resolved using Verigene® Enteric Pathogens Nucleic Acid Test (EP). 36 challenge samples (13 *Salmonella* spp., 3 *Shigella* spp., 10 *Campylobacter* spp., and 10 Shiga toxin positive *Escherichia coli*) were tested using reference strains (American Type Culture Collection) and previous patient isolates diluted to10^3^-10^4^ cfu/ml in saline then added to Sample Buffer Tube (SBT) with negative stool matrix delivered via a swab. Limit of detection testing was performed by serial 10 fold dilutions in saline then added to SBT with negative stool matrix provided via a swab.

**Results:**

A total of 272 rectal swab specimens were evaluated and 89 were positive by culture and/or MAX EBP. All discrepant results were BDM-EBP positive and culture negative. 21 of 31 (68%) of the apparent false positive BDM-EBP discrepant results resolved as positive with Nanosphere’s Verigene® EP. After resolution of the discordant results, the Positive Percent Agreement (PPA) and Negative Percent Agreement (NPA) are as follows for each target: *Salmonella* (*n* = 4) 100%, PPA and 100%, NPA; *Shigella* (*n* = 79) 100%, PPA and 95.3%, NPA; *Campylobacter* (*n* = 4) 100%, PPA and 99.6%, NPA; and Shiga toxin producing organisms (*n* = 2) 100%, PPA and 100%, NPA. 8.8% of the patient samples did not initially yield a result on the BDM-System. Upon repeat, half of the problematic samples resolved, and 4.4% of the total specimen tested did not yield a result. All organisms in the challenge samples were detected. Limits of detection for BDM-EBP testing of rectal swabs were as follows (in cfu/ml in SBT): *Salmonella*-1.44 × 10^2^; *Shigella*-5.10 × 10^0^; *Campylobacter*-1.51 × 10^1^; and Shiga Toxin-1.13 ×10^3^.

**Conclusion:**

Rectal swabs are acceptable samples for detecting *Salmonella*, *Shigella*, *Campylobacter*, and Shiga toxin using BDM-EBP.

## Background

According to the World Health Organization reports, there are nearly 1.7 billion cases of diarrheal disease worldwide every year [1, 2]. In addition, this disease is the second leading cause of death worldwide for children under 5 years of age, killing nearly 760,000 [1, 2]. Repeated bouts of diarrhea and persistent diarrheal disease disrupt intestinal function and absorption, making diarrheal disease a leading cause of malnutrition in this same age group [1, 2]. Importantly, diarrheal disease is both treatable when correctly diagnosed and preventable via proper infection control measures.

In the United States, *Salmonella, Campylobacter, Shigella, and* Shiga toxin-producing *Escherichia coli* are the most common bacterial pathogens, and they are usually associated with foodborne illnesses. Identifying the cause of diarrhea is important for the treatment of patients and for public health intervention through outbreak management.

The BD MAX™ Enteric Bacterial Panel (BDM-EBP) from BD Diagnostic Systems (Quebec, Canada) identifies *Salmonella, Campylobacter, Shigella, and* Shiga toxin-producing *E. coli* via multiplex PCR*.* The BDM-EBP is FDA-cleared to detect these four pathogens from fresh stool cultures and stool preserved in Cary-Blair. In a large, multi-centered study comparing BDM-EBP with stool culture for fresh and preserved stool specimen, the Positive Percent Agreement (PPA) and Negative Percent Agreement (NPA) were as follows: *Salmonella*−97.3% PPA, 99.8% NPA; *Shigella*−99.2% PPA, 100% NPA; *Campylobacter*−97.5% PPA, 99.0% NPA; and Shiga toxin producing organisms−100% PPA, 99.7% NPA [3]. However, rectal swabs, which are not FDA-cleared for clinical testing with the BDM-EBP, are common specimens received for stool pathogens. Rectal swabs are widely accepted to be an appropriate sample type for infants and patients that cannot pass a stool specimen [4, 5]. The application of nucleic acid amplification to this sample type would have a significant impact on diagnosis and treatment as well as understanding the epidemiology of this disease, particularly in very young children. The purpose of this study was to evaluate the ability of the BDM-EBP to detect stool pathogens from rectal swabs.

## Methods

### Clinical samples

From August 2015 to December 2015, 272 sequential rectal swab samples (dual swab; BBL CultureSwab (BD) in liquid Stuart) were collected from patients at Cincinnati Children’s Hospital Medical Center (CCHMC) for conventional bacterial culture and Shiga Toxin testing, as standard of care. One swab was plated onto BBL Trypticase Soy Agar with 5% Sheep Blood, MacConkey II Agar, Hektoen Enteric Agar, MacConkey II Agar with Sorbitol, and Campy CVA Agar (BD Diagnostic Systems, Sparks, MD, USA) and incubated following standard protocols. Colonies consistent with enteric bacterial pathogens were identified using Vitek 2 GN identification cards (bioMerieux, Durham, NC, USA), conventional biochemical reactions, MALDI-ToF, and serogrouping. After inoculating the agar plates for standard of care, the “remnant” swab was placed into a SBT, vortexed, and removed. Testing of the SBT was carried out with the BDM-EBP according to the manufacturer’s package insert: the SBT was mixed and placed on the instrument with extraction reagents and master mix, where the nucleic acid extraction, amplification, and detection occurred. Shiga-toxin EIA testing was performed on the second swab in the rectal swab collection device using MacConkey Broth (Remel, Lenexa, KS) and the Immunocard STAT! EHEC test method (Meridian, Bioscience, Cincinnati, OH) according to the manufacturer’s instructions. All SBTs were frozen at−80 °C for future testing, if needed. Prior to evaluating the performance of BDM-EBP with rectal swab specimens, a preliminary study was done to assess if inoculating swabs onto agar plates before inoculating the BDM-SBTs impacted the amount of organism present or inhibited detection by PCR. The findings showed that BDM-EBP detection of various dilutions of *Salmonella enterica* subsp. enterica serovar Typhimurium *(*ATCC 14028) was similar for swabs streaked first on HE agar plates (BD Diagnostic Systems, Sparks, MD, USA) and swabs placed directly in BDM-SBT directly, confirming that the bacterial load was not impacted for PCR testing (data not shown).

### Discordant analysis

Discordant analysis was performed on the SBT employing the Verigene® EP (Enteric Pathogen) kit on the Verigene System (Nanosphere, Northbrook, IL) using a modification of manufacturer’s protocol. The SBT was also sub-cultured to appropriate agar plates to enhance the isolation of the pathogen. The previously frozen SBT was thawed and mixed thoroughly with a vortex. 300 μl of the SBT was placed into a micro-centrifuge tube and several drops were plated onto appropriate media and incubated accordingly. Stool matrix prescreened by BDM-EBP and Verigene® EP and determined to be negative was added to the micro-centrifuge tube via a swab. The contents of the tube were mixed thoroughly and spun at 2000 rpm for 30 s. 200 μl of the SBT/stool matrix mix was added to the extraction tray. The extraction tray was capped and loaded into the Nanosphere processor with the tips, reagent tray, and Verigene® EP test cartridge where testing was performed.

### Statistical analysis

Positive percent agreement (PPA) and negative percent agreement (NPA) between BDM-EBP and culture/EIA were calculated for clinical samples for each target, as appropriate.

### Challenge samples

Thirty-six additional contrived samples (3 *Shigella* spp., 13 *Salmonella* spp., 10 *Campylobacter* spp., and 10 *E. coli* isolates known to produce Shiga toxin) were also tested. Reference strains (American Type Culture Collection) and previous patient bacterial isolates were diluted to a final density of 10^3^-10^4^ cfu/ml in saline. 10 μl of the dilution was added to a SBT. Negative stool matrix was added to the SBT via swabs and tested with the BDM-EBP kit on the BDM instrument.

### Limit of detection

To determine the limit of detection for each target, a 0.5 McFarland suspension of each organism was made: *Salmonella enterica* subsp. enterica serovar Typhimurium ATCC 14028, *Shigella sonnei* ATCC 9290, *Campylobacter jejuni* ATCC 33291, and *E. coli* ATCC 43890. Ten-fold serial dilutions of each organism were performed using saline. In triplicate, 10 μl of each dilution was plated onto appropriate agar plates and incubated at 35 °C ambient air for 24 h (42 °C, microaerophilic conditions for 72 h for *C. jejuni)*. 100 μl of each dilution was inoculated into Sample Buffer Tubes (SBTs) in triplicate. To replicate the rectal swab sample type, stool matrix prescreened with BDM-EBP and Verigene® EP and determined negative was added to each SBT by dipping the swab into the matrix then swirling in the SBT. Each SBT was tested with the BDM-EBP kit on the BDM instrument.

## Results

A total of 272 patient rectal swabs were received into the laboratory at the time of an outbreak of *Shigella* species. Of the 89 positive samples detected, 31 yielded discrepant results between culture and BDM-EBP: 28 *Shigella* sp., 1 *Salmonella* sp., 1 *Campylobacter* sp., and 1 Shiga Toxin producing organism (Table 1). All were culture negative and BDM-EBP positive.

**Table 1:** Clinical Performance of BDM-EBP with Rectal Swabs (*n* = 272)

Organism	Culture/EIA Positive/BDM- Positive	Culture/EIA Negative	
		BDM- Positive	BDM- Negative
*Shigella*	51	28	193
*Salmonella*	3	1	268
*Campylobacter*	3	1	268
Shiga Toxin^*a*^	1	1	270

^*a*^104 Single swabs received. Shiga Toxin EIA not performed

The residual SBTs for 21 of the 31 culture negative/BDM-EBP positive samples were also positive by Verigene® EP, using the modified protocol (Table 2). No additional positive samples were detected sub-culturing SBT to agar media. After resolution testing, the Positive Percent Agreement (PPA) and Negative Percent Agreement (NPA) were as follows for each target: *Salmonella* (*n* = 4) 100%, PPA and 100% NPA; *Shigella* (*n* = 79) 100%, PPA and 95.3%, NPA; *Campylobacter* (*n* = 4) 100%, PPA and 99.6%, NPA; and Shiga toxin producing organisms (*n* = 2) 100%, PPA and 100%, NPA. The assay limit of detection for simulated rectal swab specimens ranged from 5–1130 cfu/ml in SBT (Table 3), and the 4 targets were correctly detected in all of the contrived challenge specimens.

**Table 2:** Resolution of Discrepant Results using Verigene® EP

Culture Negative; BDM-EBP Positive Discrepant Specimens	Verigene Results (% of discrepant results)	
Target	Positive	Negative
*Shigella* (*n*=28)	19 (68%)	9 (32%)
*Salmonella* (*n*=1)	1 (100%)	0 (0%)
*Campylobacter* (*n*=1)	0 (0%)	1 (100%)
Shiga Toxin (*n*=1)	1 (100%)	0 (0%)

**Table 3:** Assay Limit of Detection with Simulated Rectal Swabs

Target	Limits of Detection in SBT
*Shigella* spp.	5 cfu/ml
Shiga Toxin genes *stx1/2*	1130 cfu/ml
*Campylobacter* spp.	15 cfu/ml
*Salmonella* spp.	144 cfu/ml

## Discussion

In this study, 31 rectal swabs were positive by BDM-EBP but were negative by culture, suggesting that BDM-EBP is more sensitive than culture for detecting bacterial stool pathogens from rectal swabs. While it is possible that these BDM-EBP results are false positives, this is unlikely as 68% of these discrepant samples were also found to be positive by the Verigene® EP. The ability of the BDM-EBP to detect additional positives is likely the result of improved sensitivity compared to culture of rectal swabs and this is finding is consistent with the assay limit of detection for the 4 targets, using simulated rectal swab specimens.

In a large, multi-centered study comparing BDM-EBP with stool culture for fresh and preserved stool specimen, the PPA and NPA were as follows: *Salmonella*−97.3% PPA, 99.8% NPA; *Shigella*−99.2% PPA, 100% NPA; *Campylobacter*−97.5% PPA, 99.0% NPA; and Shiga toxin producing organisms−100% PPA, 99.7% NPA [3]. The PPA and NPA between culture/Shiga toxin EIA and BDM-EBP for rectal swab specimens following resolution of discordant results are similar in the present study.

The swab transport is a critical component to the study. Cloud, et al. reports that the material at the tip of the swab can inhibit the PCR reaction [6]. Rayon or polyester is acceptable material for the PCR reaction [6]. The swab utilized within the CCHMC healthcare system is the BBL CultureSwab Dacron in liquid Stuart medium. Newer flocked swabs are designed to have more sampling area to collect more sample, as well as release more specimen into the medium. ESwab™, which utilizes the flocked swab in 1 ml Amies medium, allows for liquid testing and, thus, could be easily used on the BDM-System. However, ESwab™ is not FDA-cleared for testing with the BDM-System and must be validated by the user.

One of the limitations of this study was the low number of *Salmonella, Campylobacter* and Shiga toxin positive rectal swabs obtained. Although all the contrived specimens tested were correctly identified, true patient specimens that are positive are ideal. As rectal swabs are not an approved sample type for any multiplex gastrointestinal panel, discordant analysis relied on using Nanosphere’s Verigene® EP to test the SBT in an off-label manner. Preferably, an alternate PCR with bidirectional amplicon sequencing would be used to resolve discrepant results, but this testing was beyond the scope of this study [3].

The initial rate for patient specimens where no result was obtained was 8.8%. Upon a single repeat, 50% of the problematic specimens resolved, and the final percentage of total specimens tested with no result was 4.4%. Review of a large multi-center study indicated initial unresolved rates of 5.0% with some sites reporting initial unresolved rates of 7.7% and 10%. Upon repeat of the initial SBT, the unresolved rate dropped to 1.3% with the same sites reporting repeat unresolved rates of 1.6% and 4.5% respectively. This study also indicated unpreserved specimen had higher unresolved rates than preserved specimen. Dilution of the stool matrix in Carey-Blair thus reducing inhibitory substances was given as a plausible explanation for the differing unresolved rates [3].

## Conclusion

Although stool is the optimal specimen for identifying enteric bacterial pathogens, PCR from swabs is more sensitive than culture from swabs, and rectal swabs can be used for testing with BDM-EBP for detection of *Salmonella* spp., *Shigella* spp., *Campylobacter* spp., and Shiga toxin 1 and 2 genes. Additionally, BDM –EBP PCR results are available 34–47 h sooner that with conventional cultures from rectal swabs [7]. Early detection will aid in epidemiology and managing the spread of these bacterial pathogens. The application of nucleic acid testing with the BDM-EBP to rectal swabs has the potential to increase pathogen identification of diarrheal disease. This is particularly true among the very young, the population most at risk for mortality from untreated diarrheal disease.

### IRB Review

Swab samples were remnant, discarded, de-identified samples. This study was determined to not be human subjects research and, therefore, exempt from the oversight of the Institutional Review Board.

## Abbreviations

EBP: Enteric Bacterial Panel
SBT: Sample Buffer Tube
EP: Enteric Pathogen
PPA: Positive Percent Agreement
NPA: Negative Percent Agreement
